# How do couples influence each other’s physical activity behaviours in retirement? An exploratory qualitative study

**DOI:** 10.1186/1471-2458-13-1197

**Published:** 2013-12-18

**Authors:** Inka Barnett, Cornelia Guell, David Ogilvie

**Affiliations:** 1 MRC Epidemiology Unit and UKCRC Centre for Diet and Activity Research (CEDAR), Institute of Public Health, University of Cambridge, Cambridge, UK; 2 Institute of Development Studies (IDS), University of Sussex, Brighton BN1 9RE, UK; 3 Faculty of Medical Sciences, University of West Indies, Bridgetown, Barbados

**Keywords:** Retirement, Physical activity, Qualitative study

## Abstract

**Background:**

Physical activity patterns have been shown to change significantly across the transition to retirement. As most older adults approach retirement as part of a couple, a better understanding of how spousal pairs influence each other’s physical activity behaviour in retirement may help inform more effective interventions to promote physical activity in older age. This qualitative study aimed to explore and describe how couples influence each other’s physical activity behaviour in retirement.

**Methods:**

A qualitative descriptive study that used purposive sampling to recruit seven spousal pairs with at least one partner of each pair recruited from the existing EPIC-Norfolk study cohort in the east of England, aged between 63 and 70 years and recently retired (within 2-6 years). Semi-structured interviews with couples were performed, audio-recorded, transcribed verbatim and analysed using data-driven content analysis.

**Results:**

Three themes emerged: spousal attitude towards physical activity, spouses’ physical activity behaviour and spousal support. While spouses’ attitudes towards an active retirement were concordant, attitudes towards regular exercise diverged, were acquired across the life course and were not altered in the transition to retirement. Shared participation in physical activity was rare and regular exercise was largely an individual and independent habit. Spousal support was perceived as important for initiation and maintenance of regular exercise.

**Conclusions:**

Interventions should aim to create supportive spousal environments for physical activity in which spouses encourage each other to pursue their preferred forms of physical activity; should address gender-specific needs and preferences, such as chances for socialising and relaxation for women and opportunities for personal challenges for men; and rather than solely focusing on promoting structured exercise, should also encourage everyday physical activity such as walking for transport.

## Background

The transition to retirement has been identified as a critical time for the promotion of physical activity. Previous research including a systematic review of quantitative studies suggests that recreational physical activity and exercise increases in retirement, whereas the impact on overall physical activity levels is less clear [1-3]. To better understand the underlying reasons for these changes, we recently conducted a systematic review of qualitative evidence on the experience of physical activity in retirement [4]. Expected health benefits, lifelong physical activity patterns and opportunities for socialising, personal challenge and regular physical activity as part of a new routine emerged as some of the key motives for an increase in recreational physical activity after retirement. The review also highlighted that older adults often had broad concepts of physical activity that went far beyond ‘exercise’ and included a variety of domestic chores. A major shortcoming of existing qualitative and quantitative evidence is that all studies included in these reviews adopted an individualistic approach to physical activity in retirement; no study considered the family context or, more specifically, the influence a spouse might have. Spousal influences are likely to become more important following retirement, as partners often spend considerably more time together and social networks are reduced due to a loss of work-related contacts [5]. Spouses can influence each other’s health behaviours including physical activity in different ways. For example, a partner might motivate health-enhancing behaviours [6-8] or initiate or increase health-damaging behaviours [9].

A number of studies have investigated the association between marital status and changes in physical activity in newlyweds or young couples [10,11], whereas few studies have examined physical activity in longer-term marriages. The findings of those studies that exist are inconsistent, with some reporting higher physical activity levels in married older couples [12] and others finding no association [13,14]. In intervention studies that aimed to increase physical activity levels among older adults, participants who joined programmes together with their spouses were more likely to adhere to the intervention than those who took part on their own [15,16].

Given that most people retire as part of a couple [17], a better understanding of how intimate partners influence and shape each other’s physical activity behaviour could provide important information for the design and targeting of future interventions. Despite considerable efforts and campaigns to promote physical activity, physical activity levels remain inadequate for good health among older adults [18-21]. Insufficient physical activity has been shown to increase the risk of chronic diseases such as cardiovascular disease, type 2 diabetes, hypertension and several cancers as well as functional limitations, depression and premature death in the elderly [21-23]. This qualitative study aimed to explore and describe how cohabitating partners, of whom at least one has recently retired, influence each other’s physical activity behaviour.

## Methods

A qualitative descriptive approach as described by Sandelowski [24,25] was used because it allowed a comprehensive description and exploration of couples’ shared experiences of physical activity in retirement [26]. Spouses were interviewed together to provide a better understanding of how partners may influence each other’s physical activity behaviours [27,28].

### Participant selection and setting

As suggested for a qualitative descriptive study, purposive sampling was employed to allow maximal variation between couples [29]. At least one member of each couple was recruited from the existing European Prospective Investigation into Cancer and Nutrition (EPIC)-Norfolk study, a cohort study of initially 25,639 men and women selected from the general population aged 45-79 years [30]. The EPIC-Norfolk study is based in Norfolk, a largely rural county in the East of England with low outward migration among middle-aged adults [30]. Recruitment and baseline assessment of the EPIC-Norfolk cohort took place between 1993 and 1997 through general practitioner surgeries. Participants completed questionnaires on their diet, lifestyle and health after 18 months, 3 years, 10 years and 13 years and also attended two health checks. For this study, we recruited at least one member of each couple from among the ~10,000 EPIC-Norfolk participants who attended the 13-year follow up and invited them to take part together with their spouses. Recruiting one spouse from an existing cohort enabled us to select a diverse sample in terms of area of residence and occupational background. The selection criteria were that participants had to be married or cohabitating with a partner, aged 60-70 years, recently retired (within the past 2-6 years) and not retired due to ill health. We also excluded adults who had a medical condition that could prohibit even low-intensity physical activity (such as severe cardiovascular conditions, cancers or acute orthopaedic problems). As our aim was to explore a range of influences a partner could have on physical activity formation, we did not apply any exclusion or inclusion criteria to the partners of EPIC participants. Our sample could therefore include partners who were still employed or those with physical limitations.

We approached 22 potential couples, of whom eight replied to the invitation and seven couples (14 participants) aged between 63 and 70 years took part (Table 1). The time frame did not allow for additional recruitment but the analysis showed that couples shared very similar experiences and it was decided that saturation was reached. In two of the participating couples, both partners were EPIC-Norfolk participants. Pathways and lengths of retirement varied between participants, and some spouses were still in part-time employment. All couples lived together and had been married for at least 25 years. Participants’ current physical activity behaviour was assessed during the interview by asking whether they ‘do any physical activity nowadays’.

**Table 1:** Characteristics of the seven participating couples

**Couple**	**Pseudonym**	**EPIC cohort participant**	**Age**	**Former occupation**	**Time in retirement**	**Physical activity behaviour**
Couple 1	Arnold	No	66	Ironworker	2 years	Daily dog walking, regular gardening, regular DIY, walking for transport
	Norma	Yes	64	Healthcare worker	4 years	Daily dog walking, occasional swimming, daily cycling, regular gardening, occasional child care, occasional line dancing, walking for transport
Couple 2	Stan	Yes	63	Military/office manager	5 years	Regular recreational walking, occasional swimming, occasional cycling, regular gardening, occasional child care, walking for transport
	Mary	Yes	65	Retail manager	2 years	Regular recreational walking, occasional cycling, regular weight training, regular gardening, occasional child care, walking for transport
Couple 3	Ralph	Yes	63	Military/administrative assistant	Gradual retirement	Occasional recreational walking, occasional gardening
	Gwen	Yes	65	Administrative assistant	5 years, does regular voluntary work	Occasional recreational walking, regular swimming, regular aerobic classes, regular gardening, regular walking for transport
Couple 4	Terry	No	68	Gardener	Partly retired	Regular running, regular race cycling, regular exercise classes, regular weight training, cycling for transport
	Louise	Yes	65	Administrative assistant	2 years	Regular recreational walking, occasional cycling, occasional gardening
Couple 5	Peter	Yes	66	Head teacher	4 years, regular voluntary work	Daily dog walking, occasional DIY, regular childcare, regular care for elderly parents
	Jill	No	64	Secretary	5 years	Daily dog walking, regular dog agility classes, regular childcare, regular care for elderly parents
Couple 6	John	No	70	Gardener	Partly retired	Regular gardening (part-time occupation), regular recreational walking, regular childcare, regular walking for transport
	Pamela	Yes	65	Administrative assistant	4 years	Regular treadmill exercise at home, regular childcare, regular walking for transport
Couple 7	Graham	No	67	Printer	4 years	Regular recreational walking, regular gardening, regular golfing, walking for transport
	Kate	Yes	65	Pharmacy assistant	5 years	Regular recreational walking, regular keep-fit classes, regular gardening, walking for transport

### Data collection

Semi-structured interviews with open-ended questions were used to obtain in-depth understanding of how couples influence each other’s physical activity behaviours. Initially we had planned a combination of both joint and separate interviews with the couples. Joint interviews can elicit a more complete account of couples’ joint experiences and allow the observation of the dynamics and power relationships between partners that might affect negotiations of physical activity and other behaviours [31,32]. Separate interviews allow each partner to express beliefs and perceptions more freely and thereby establish a ‘sense of equity’ between spouses [33], which is perceived as particularly important if behaviours diverge between spouses [28,34]. We started with a joint interview to build up rapport and increase participants’ ease and confidence in the interview situation [35,36]. During the first three joint interviews we observed that spouses actively encouraged each other to share information and that only minimal prompting by the interviewer was necessary. Partners were very aware of each other’s attitudes, beliefs, preferences and dislikes and commented on their own as well as their partners’ behaviours without restraint. It became apparent that we could discuss couples’ influence structures extensively and freely during the joint interviews. We therefore concluded that separate interviews would not yield any further information. A common challenge in joint interviews can be that one partner dominates the conversation and acts a the ‘spokesperson’ for the couple [37]. In two of our interviews, one partner took on this role at the beginning of the interview. The interviewer gently encouraged the less dominant partner with specific questions, but generally encouraged the couple to develop their own rules of participation in the interview.

The flexible topic guide covered questions concerning the experience of physical activity in retirement and how spouses might influence each partner’s physical activity behaviours, having first been piloted with a recently retired couple in a similar age range and slightly modified as a result (Additional file 1). As we were interested in participants’ perceptions and concepts of physical activity in retirement, we did not provide a specific definition of physical activity but asked them to define physical activity in their own terms. Consequently, participants included a wide range of activities of daily living such as exercise and leisure time activities, activities in and around the home, work-related activity and active travel such as walking or cyclingi for transport. Participants were given the opportunity to speak freely and to raise additional topics and ask questions at the end of the interview [38]. All participants gave written informed consent to participate in the study, which was approved by the Norfolk Research Ethics Committee and adhered to the RATS guidelines on qualitative research [39].

Interviews were conducted by one of the authors (IB) in the participants’ homes and lasted for between 45 and 60 minutes. Interviews took place in two waves, with the first two interviews in November and December 2011 and a further five in January 2012. The time between the waves was used by the first author (IB) to reflect together with the second (CG) on initial findings and emerging themes that were then followed up in the second wave. This peer-debriefing also facilitated reflexivity and exploration of alternative explanations. Interviews were audio-recorded on a digital voice recorder (with participants’ permission) and transcribed verbatim. Throughout the interviews a research diary was kept to note down pre- and post-interview reflective thoughts, observations and impressions.

### Data analysis

As recommended for qualitative descriptive studies, a data-driven content analysis approach was employed [24]. In accordance with the aims of the study, the analysis was focussed on spousal influences on each other’s physical activity behaviour in retirement. Analysis was conducted concurrently with the data collection as described previously. The analysis started with a check of the transcripts of the interviews to ensure accuracy [38]. Each transcript was then read several times to permit familiarisation with the data and to identify initial patterns [40]. Three transcripts were read carefully line-by-line and initial codes were developed [40,41]. After this open coding, an initial coding scheme was developed that guided the coding of the remaining four transcripts. In the process, codes were repeatedly modified or combined and parts of transcripts were recoded. Codes were then sorted into emerging categories based on relations and interlinks. These categories were further combined into hierarchical structures if possible [42]. Open codes were summarised into emerging themes [26]. Data from the field notes were used to further inform the development of codes and categories. To increase the rigour of the analysis, the second author (CG) analysed three transcripts independently and combined results with the first author. This investigator triangulation provided different perspectives on the textual data and ensured a deeper understanding of the data. There were very high levels of agreement in the coding of the main categories and high levels of agreement in the detailed coding between the two researchers, with no instances of significant disagreement.

All identifying information (names, locations, person-identifiable information, etc.) were removed from quotes to ensure anonymity and pseudonyms were used. Open-source WEFT QDA software was used to assist with coding, cross-referencing, storage and retrieval. This study adheres to the RATS guidelines on qualitative research [43].

## Results

**Figure 1:**
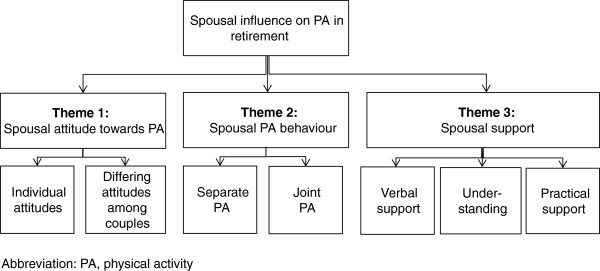
Emerging themes from the analysis of the seven interviews.

Three themes that captured spousal influences on each other’s physical activity behaviours emerged from the analysis of the 130 pages of transcripts: spousal attitude towards physical activity, spouses’ physical activity behaviour and spousal support (see Figure 1).

### Attitude towards physical activity

#### Individual attitude towards physical activity

All participants believed that it was important to remain active in retirement. Mary and Kate described the consensus of the group:

Well I think you have to do something, don’t you, when you work sort of five days a week and you’re active and you’re doing all that, and then nothing, you’ve got to do something. (Mary)

I must, when I retired I did sort of say to myself that I would not watch television during the day because I think if you start that that’s something that you just sit and watch television and I sort of made a conscious effort that I would not do that. (Kate)

The majority of participants perceived themselves to be sufficiently active with daily chores such as gardening, housework and minding grandchildren, as expressed by Arnold:

Although I’ve been finished about two years and I got plenty to do, I got like this, in this house, like decorating, gardening, (sighs) looking after a car, like, you know, anything, you know, but I’ll keep myself busy. (Arnold)

While most participants walked (more or less frequently) for recreational purposes, other more vigorous activities were less frequently reported. For those who regularly exercised, participation had become a well-established and life-long habit and source of pleasure which they had always attempted (at times more or less successful) to integrate in their lifestyles. Gwen reflected:

I think almost since we were married and our children were young, I’ve nearly always been to a keep-fit class of some form or another, different ones over the years. Swimming, I always enjoyed when I was younger. I think I then went for a spell without doing any swimming and, you know, I’d worked part-time and I found I had… you know, my boys were sort of growing up and I had a bit more time. I just enrolled with this leisure club, and that was many years ago now, and, you know, I’ve been ever since. (Gwen)

#### Differing attitudes towards physical activity among couples

Perceptions of active lifestyles covered a range of activities, from ‘not watching television during the day’ to structured exercise. While spouses agreed on the importance of an active lifestyle in retirement, opinions regarding regular exercise diverged and were often in opposition, with one partner being less interested or uninterested in regular exercise. On the other hand, one couple described how her (Norma’s) positive attitude towards regular exercise encouraged him (Arnold) to start thinking more about his own (unhealthy) lifestyle after retirement and to introduce small changes (e.g. walking rather than using the car):

You used to hop in the car quite… like that was like a thing with you, you’d get the car out and go when you worked away, weekends, that would be more, but now you would walk round Peter’s or walk round Martin’s. I mean you never used to do that, did you? (Norma)

Well, yeah, and she’s one for… always one for healthy living……and healthy foods. (Arnold)

Several less-active participants viewed regular exercise purely as an ‘interest’ or ‘hobby’ of their spouse which they had never shared. Arnold stated how his wife had always enjoyed active leisure time pursuits whereas he enjoyed different sedentary activities:

So we do things together, if we’ve got something we do different, like her with her line dancing or swimming, for me with my football [watching on television] or whatever […] (Arnold)

A few men were also convinced that they did not need to do regular exercise as they were ‘fit enough to do exercises’ (Arnold) and had not ‘notice any particular slacking of pace really, so it’s still there I think’ (Peter). Their spouses agreed that their husbands might have ‘far more stamina’ or were ‘for your age, you’re quite agile’ (Pamela). Nevertheless, one wife (Mary) also reminded her husband that his perceptions of his own fitness levels might be outdated and based on a younger version of himself.

One man (Peter), while believing that it was important to remain active in old age, also expressed doubts about the health benefits of exercise:

Yeah, you don’t know. I’ve often wondered whether you’d be better for doing more exercise, but then I see people who do and they’ve got bad legs, hips and all sorts. I don’t know, it’s difficult to know what’s best for you, and we tend to do what we’re just comfortable with, don’t we basically? (Peter)

While his wife reiterated his concerns, her own favourable attitude towards exercise and her regular exercise routine remained unchanged.

The more active male participants often associated physical activity with competitiveness or a challenge. Engaging in any physical activity or exercise without achieving a goal (e.g. testing their personal fitness level, or beating others in a competition) was perceived as ‘wasting your time’ (Terry) and ‘wouldn’t do anything for me’. (Stan)

Their wives did not share this competitive attitude and participated in physical activity only for recreational purposes, as noted by Louise:

I mean I’m not, I’ve no competitive spirit whatsoever, I’m not a competitor in any sense, and I like to be outside if I’m exercising to be honest, I’m much happier in the fresh air so you know […] (Louise)

These women recalled how their husbands’ competitive behaviours had made past attempts at joint physical activities (e.g. recreational walks) a frustrating and, for one couple, a never-to-be-repeated experience.

### Spouses’ physical activity behaviour

#### Separate physical activity

In accordance with their diverse attitudes and interests, spouses engaged in different (if any) forms of regular physical activity. Women tended to be more engaged in regular exercise than their husbands, mainly because they continued their established exercise routines after retirement. A few participants felt encouraged by the physical activity behaviour of their spouses to become more active themselves. For some this meant an increase in purposeful physical activity such as walking for transport; for others, adoption of regular exercise. All of those who felt motivated had a long-standing interest in and previous history of regular exercise themselves, but had stopped after the transition to retirement or due to work commitments. Other participants who did not feel motivated by their spouses’ exercise behaviour described how they had never enjoyed exercise and only exercised when it was required of them (e.g. at school, in the army or in a previous occupation).

While we did not find any instances of spouses attempting to actively restrict each other’s physical activity participation, one inactive husband (Ralph) suggested that the harmony within their couple relationship would be disturbed if his wife started to engage in competitive sports instead of non-competitive recreational activities. His wife (Gwen) was aware that ‘there would be more tension’ and assured him:

No, I wouldn’t enjoy competitive sport. I don’t think I ever have. (Gwen)

In one couple (Terry and Louise), the spouses had very different physical activity behaviours, with Terry being a keen amateur athlete and Louise’s main exercise consisting of low-intensity recreational walks. His daily training sessions were a source of frequent frustration for his wife Louise:

[…] it interferes with something that matters a bit but generally not, no, you get used to it. (Louise)

Terry did not sympathise and stressed that

You [wife] learned very early on thou shalt not become between a man and his sport, don’t do it, if you want a relationship to work you don’t do it.

In a few couples both spouses engaged in regular but different forms of exercise. Separate participation provided each partner with personal ‘space’ and time away from the spouse after retirement, which was valued as ‘24/7 can be, get a bit… [much]’. (John)

It also enabled both of them to socialise within the same gender group, as expressed by Mary:

[…] I think also it’s good because we’re together all the while and he has male company on that day as well and they talk about men things, which is good as well, isn’t it, than being in female company. (Mary)

Personal time could also be achieved through sedentary leisure-time pursuits, as pointed out by Jill:

Well you think the dogs are my hobby really and I also, in the summer, I like gardening, I quite enjoy that and you continue to do the magistrates don’t you and that’s your hobby and we both felt that we wanted to keep something for ourselves. (Jill)

#### Joint physical activity

Shared physical activity was rare owing to diverse interests and different personal goals and ambitions, as stated by Ralph and Stan.

Yes, I’ve got nothing against. I mean, if we both like something, then we’ll probably do it together, like going on a walk, but that’s probably the only thing because you don’t play any sports as such, do you? (Ralph)

But as far as the walking is concerned, I don’t choose walks that are like round a road circuit, I choose walks that will give me some hills and some climbing over fences and that sort of thing. Yeah, we climb over all sorts. (Stan)

However, some couples walked together more frequently after the transition to retirement because they had more time flexibility and it offered opportunities to spend quality time together. Stan and Mary fondly described their weekly walk as their ‘courting day’ or ‘date day’ which offered them the chance ‘to do things together’.

Holidays were an exception from everyday physical activity patterns for most couples, as Louise noted:

Even when we’re away on holiday together we walk together you know, we go to places and climb hills and walk and things but that’s different, that’s not a, you know, daily basis is it dear? (Louise)

### Spousal support

The data indicated three categories of spousal support for physical activity: verbal encouragement, understanding and practical support.

#### Verbal encouragement

Several physically active women verbally encouraged and in some cases ‘nagged’ their partners to be more active after the transition to retirement, as noted by Stan:

Yeah, but having said that, yes I did slow down for about six months, and I would say that I am more sedentary now than I would like to be, but, you know, my wife says I’ve got to switch that telly off more often and get out and do something. So I do admit to that, I admit to being a bit more sedentary than I ever was. (Stan)

The husbands who were encouraged in this way described how their partners’ frequent comments eventually motivated them to change their behaviour and ‘walk more’ (Arnold) or initiate regular exercise. Stan reflected:

Yeah, and I suppose the constant bombardment for me to do something motivated me to do it…. (Stan)

#### Understanding

Providing support by understanding their spouses’ interest in exercise was commonly described by both active and less active partners. Stan and Arnold explained:

I always thought you [wife] enjoyed swimming, so that didn’t surprise me you want to go there and you. (Stan)

[…] that’s something she [wife] liked doing, what I wouldn’t bother [her] to do, you know. (Arnold)

The wife of the amateur athlete Terry explained how she had always supported her husband by understanding and not interfering with his lifestyle choice.

[…] not ever stopped in his way so that’s encouragement if you like, so. (Louise)

She felt that her husband did not always appreciate and acknowledge her understanding. Terry, in contrast, described how he always wished his wife would take more interest in his sporting ambitions and accompany him to competitions. The couples’ divergent attitudes towards exercise became further apparent in her (mis?)interpretation of his attempts to encourage her to be physically active. Louise felt pressurised and controlled and was reluctant to do anything more ‘serious’ than recreational walking ‘for pleasure’.

#### Practical support

Men frequently provided practical social support such as transport or help with household chores to allow their partners the necessary time to do exercise. Jill described in the following quote how her husband’s support allowed her to participate regularly in agility training with her dog, an exercise Peter did not attend himself:

Yeah. Unfortunately the agility classes have actually coincided with Sunday mornings, so I have to, I get up, and then between us we rush round sort of doing vegetables and so on, then we go for a couple of hours to the agility, come back and then finish it all off and then we get it all done don’t we (laughs). (Jill)

## Discussion

While there is a large body of literature on physical activity in retirement, this is the first study that explores how spouses influence each other’s physical activity behaviour in retirement. We found that each couple had established unique influence structures with regards to physical activity and exercise which were shaped by each spouse’s interests, motivations and previous history of physical activity. All couples were concordant in their belief that it was important to maintain an active lifestyle in retirement, corroborating the findings from previous individual-level qualitative research on physical activity in retirement [4,44-46].

However, most spouses diverged (to varying degrees) in their attitudes towards physical activity, in particular in the form of regular structured exercise. This was reflected in their different actual exercise behaviours. Less-active spouses often perceived exercise only as a ‘hobby’ they had never enjoyed and only reluctantly taken part in, for example at school or in the army. Previous qualitative research suggests that older adults’ lifetime experiences of and dependence on physically strenuous manual work might explain the low value placed on exercise or other recreational physical activities [4,46]. More ‘purposeful’ activities such as dog-walking seemed to appeal more in these cases. In contrast, more-active spouses valued regular exercise as an essential and lifelong component of an active lifestyle and source of enjoyment. A positive attitude and enthusiasm for exercise across the life course has been recognised as an important motivator for maintenance of regular exercise in advanced age [47] and also emerged as one of the key themes in the qualitative systematic review on the experience of physical activity in retirement mentioned previously [4].

Rather than being shared between spouses, physical activity and exercise behaviour was often gendered, with spouses following male and female perceptions and preferences (e.g. regarding competition or recreation). Accordingly, spouses were relatively independent in their behaviour and engaged in separate activities. Individual unstructured activities such as walking and exercise participation were treasured, as they offered opportunities for personal time in retirement, a benefit also highlighted by the retired members of a fitness centre in a previous study [48]. Several participants were motivated by their spouses’ regular exercise routines to become more active themselves, also confirming findings from previous research [49-51]. Using data from the Health and Retirement Study (HRS), Falba and Sindelar [52] found that spouses with regularly physically active partners were more likely to initiate exercise themselves in a five-year follow-up period. Similarly, Frank et al. [53] reported that a partner’s modelling of favourable health behaviours was an effective strategy to motivate health behaviour change in their spouse. While the experiences of some couples in this study were in accordance with these findings, we found that only spouses who had their own history of ‘voluntary’ physical activity participation were receptive to spousal role modelling with regards to exercise.

While our participants’ engagement in physical activity occurred relatively independently of their partners, spouses were nonetheless an important source of social support. Wives’ verbal encouragement seemed to be most successful in initiating an increase in physical activity in their husbands after the transition to retirement, whereas spousal support in the form of understanding and practical help facilitated the maintenance of physical activity behaviour in retirement and throughout married life. The positive effect of spousal support on health-promoting behaviours among older adults has been emphasised repeatedly [15,49,54,55], although most studies have predominantly focussed on the effect of verbal encouragement. It has been suggested that social support might indirectly affect spouses’ physical activity behaviour by increasing their capacity for self-regulatory behaviour and increasing self-esteem and self-efficacy [56-58]. However, we also found that spousal support was not always appreciated, but was sometimes interpreted as pressure or unwanted social control by the less-active spouse, with negative consequences on motivation and behaviours. Hong et al. [59] made similar observations in their study on exercise support among cardiac patients and their partners. In this study spousal support was only effective among couples with similar physical activity levels, whereas in couples with different physical activity levels the less-active partners often deliberately disregarded encouragement they received from their active partners.

There are several reasons why the findings of this study should be interpreted with caution. First, the recruitment strategy used might have introduced some selection bias. For example, the participant/s who were already taking part in the EPIC-Norfolk cohort study might have been more interested in health topics in general and more physically active then the general population of retirees. However, as their spouses were not necessarily participants in the EPIC-Norfolk study, and physical activity behaviour varied greatly between couples, our sample as a whole was not composed exclusively of highly-active participants. Physical activity behaviour also diverged greatly between spouses in both of the couples that comprised two EPIC- Norfolk participants. Second, the relatively small sample size might limit the conclusions that can be drawn. Nevertheless, the couples who participated described a variety of experiences with retirement and physical activity and provided a deeper insight into how spouses influence each other with regard to physical activity. Third, in this study none of the spouses had retired at the same time as their partners. Women had retired earlier and had already had some time to adjust to retirement and establish new routines before their husband retired. This might have helped their husband’s adjustment to retirement, although previous research suggests that spousal influence on such adjustment might be limited [5]. Fourth, none of the participants in this study had retired due to ill-health or suffered from any severe medical conditions. Ill-health in one partner might affect both partners’ abilities to be physically active; this could be an avenue for further research. The personal characteristics and professional background of an interviewer have been shown to influence qualitative interview findings [60]. All interviews were conducted by a young, female researcher with a background in physical activity research. The interviewer made clear to the participants that she had no medical background and could not provide recommendations related to medical conditions and physical activity. She stressed that she was interested in couples’ views and experiences of physical activity. Participants seemed to feel relaxed and at ease with the interview situation and said that they enjoyed talking to ‘the young woman from the University. Fifth, while all couples seemed very comfortable in the interview and openly discussed and criticised each other’s physical activity behaviour, additional separate interviews might have drawn out some additional information including further negative comments on the spouses’ physical activity behaviour. Lastly, this study was also limited in terms of ethnic diversity, highlighting a need for further research with larger and more diverse samples. However, the varied sample — including participants with different occupational backgrounds and pathways to retirement — nevertheless provided novel insights into how couples influence each other’s physical activity behaviour in retirement.

## Conclusions

We found that attitudes towards physical activity diverged between spouses and that each partner’s physical activity behaviours reflected his or her individual and independent preferences and habits. Nevertheless, spousal physical activity behaviours and spousal support can play an important role in promoting adoption and maintenance of regular exercise and physical activity in retirement. This study also highlights the importance of lifelong exercise habits as a determinant of physical activity in retirement as well as a precondition for receptiveness and responsiveness to spousal influences on physical activity.

Our findings tentatively suggest one possible intervention strategy based on encouraging spouses to support each other in pursuing their own preferred forms of physical activity. Based on our findings and those of previous research [4], physical activity interventions targeting the retired population might also be more effective if they were to address gender-specific needs and preferences, such as chances for socialising and relaxation for women and opportunities for personal challenges for men. Given that older adults without lifelong exercise habits might be less open to adopting regular exercise, interventions should not solely focus on promoting structured exercise, but also on encouraging everyday physical activity (for example through walking for transport) while taking busy post-retirement lifestyles into consideration.

## Competing interests

The authors declare that they have no competing interest. No financial disclosures were reported by the authors of this paper.

## Authors’ contributions

IB specified the research question and designed and executed the qualitative study, analysed the interviews and drafted the manuscript. CG made substantial contribution to overall research design and the interview analysis. CG and DO contributed to the specification of the research question and the writing of the manuscript. The corresponding author confirms full access to all aspects of the research and writing process, and takes final responsibility for the paper. All authors read and approved the final manuscript.

## Pre-publication history

The pre-publication history for this paper can be accessed here:

http://www.biomedcentral.com/1471-2458/13/1197/prepub

## Supplementary Material

Additional file 1 Topic guide for interview: physical activity of couples in retirement. Click here for file

## References

[B1] Barnett I van Sluijs EMF Ogilvie D Physical activity and the transition to retirement: a systematic review Am J Prev Med 2012 13 3 1 8 22898127 10.1016/j.amepre.2012.05.026 PMC3830178

[B2] Lahti J Laaksonen M Lahelma E Rahkonen O Changes in leisure-time physical activity after transition to retirement: a follow-up study Int J Behav Nutr Phys Act 2011 13 36 1 8 21513555 10.1186/1479-5868-8-36 PMC3094268

[B3] Sjösten N Kivimäki M Singh-Manoux A Ferrie JE Vahtera J Change in physical activity and weight in relation to retirement: the French GAZEL Cohort Study BMJ Open 2012 13 1 1 13 10.1136/bmjopen-2011-000522 PMC3277904 22318663

[B4] Barnett I Guell C Ogilvie D The experience of physical activity and the transition to retirement: a systematic review and integrative synthesis of qualitative and quantitative evidence Int J Behav Nutr Phys Act 2012 13 9 1 10 10.1186/1479-5868-9-97 PMC3463454 22897911

[B5] Van Solinge H Henkens K Couples’ adjustment to retirement: a multi-actor panel study J Gerontol B Psychol Sci Soc Sci 2005 13 1 11 20 10.1093/geronb/60.1.s11 15643041

[B6] Rook KS Giles H, Coupland N, Wiemann JM Social networks as a source of social control in older adults’ lives Communication, Health, and the Elderly, Volume 1 1990 Manchester: Manchester University Press 45 63

[B7] Umberson D Gender, marital status and the social control of health behavior Soc Sci Med 1992 13 8 907 917 10.1016/0277-9536(92)90259-S 1604380

[B8] Wilson SE The health capital of families: an investigation of the inter-spousal correlation in health status Soc Sci Med 2002 13 7 1157 1172 10.1016/S0277-9536(01)00253-2 12365528

[B9] Stimpson JP Masel MC Rudkin L Peek MK Shared health behaviors among older Mexican American spouses Am J Health Behav 2006 13 5 495 502 10.5993/AJHB.30.5.6 16893312

[B10] Hull EE Rofey DL Robertson RJ Nagle EF Otto AD Aaron DJ Influence of marriage and parenthood on physical activity: a 2-year prospective analysis J Phys Act Health 2010 13 5 577 583 20864752 10.1123/jpah.7.5.577 PMC3124092

[B11] King AC Kiernan M Ahn DK Wilcox S The effects of marital transitions on changes in physical activity: results from a 10-year community study Ann Behav Med 1998 13 2 64 69 10.1007/BF02884450 9989310

[B12] Pettee KK Brach JS Kriska AM Boudreau R Ayonayon HN Influence of marital status on physical activity levels among older adults Med Sci Sports Exerc 2006 13 3 541 552 10.1249/01.mss.0000191346.95244.f7 16540843

[B13] Booth ML Owen N Bauman A Clavisi O Leslie E Social-cognitive and perceived environment influences associated with physical activity in older Australians Prev Med 2000 13 1 15 22 10.1006/pmed.2000.0661 10896840

[B14] King AC Castro C Wilcox S Eyler AA Sallis JF Brownson RC Personal and environmental factors associated with physical inactivity among different racial–ethnic groups of US middle-aged and older-aged women Health Psychol 2000 13 4 354 564 10907654 10.1037//0278-6133.19.4.354

[B15] Gellert P Ziegelmann JP Warner LM Schwarzer R Physical activity intervention in older adults: does a participating partner make a difference? Eur J Ageing 2011 13 3 1 9 10.1007/s10433-011-0193-5 PMC5547339 28798651

[B16] Wallace J Raglin J Jastremski C Twelve month adherence of adults who joined a fitness program with a spouse vs without a spouse J Sports Med Phys Fitness 1995 13 3 206 213 8775648

[B17] United Nations DoEaSA Population Division World Marriage Data 2008 New Youk: United Nations 2009

[B18] Craig R Mindell J Hirani V Unit JHS Research NCfS: Health survey for England 2008: physical activity and fitness: National Centre for Social Research with permission of The NHS Information Centre 2009

[B19] Tucker JM Welk GJ Beyler NK Physical activity in US adults: compliance with the physical activity guidelines for Americans Am J Prev Med 2011 13 4 454 461 10.1016/j.amepre.2010.12.016 21406280

[B20] King AC Rejeski WJ Buchner DM Physical activity interventions targeting older adults Am J Prev Med 2004 13 4 316 333 10.1016/s0749-3797(98)00085-3 9838975

[B21] Taylor A Cable N Faulkner G Hillsdon M Narici M Van Der Bij A Physical activity and older adults: a review of health benefits and the effectiveness of interventions J Sports Sci 2004 13 8 703 725 10.1080/02640410410001712421 15370483

[B22] Nelson ME Rejeski W Blair SN Duncan PW Castaneda-Sceppa C Physical activity and public health in older adults: recommendation from the American college of sports medicine and the American heart association Med Sci Sports Exerc 2007 13 8 1435 1450 10.1249/mss.0b013e3180616aa2 17762378

[B23] Win S Parakh K Eze-Nliam CM Gottdiener JS Kop WJ Ziegelstein RC Depressive symptoms, physical inactivity and risk of cardiovascular mortality in older adults: the cardiovascular health study Heart 2011 13 6 500 505 10.1136/hrt.2010.209767 21339320 PMC3044493

[B24] Sandelowski M Focus on research methods-whatever happened to qualitative description? Res Nurs Health 2000 13 4 334 340 10.1002/1098-240X(200008)23:4<334::AID-NUR9>3.0.CO;2-G 10940958

[B25] Sandelowski M What’s in a name? Qualitative description revisited Res Nurs Health 2010 13 1 77 84 20014004 10.1002/nur.20362

[B26] Magilvy JK Thomas E A first qualitative project: qualitative descriptive design for novice researchers J Spec Pediatr Nurs 2009 13 4 298 300 10.1111/j.1744-6155.2009.00212.x 19796329

[B27] Allan G A note on interviewing spouses together J Marriage Fam 1980 13 1 205 210 10.2307/351948

[B28] Eisikovits Z Koren C Approaches to and outcomes of dyadic interview analysis Qual Health Res 2010 13 12 1642 1655 10.1177/1049732310376520 20663940

[B29] Patton MQ Qualitative research and evaluation methods, Volume 3 2002 London: Sage Publications, Inc

[B30] Day N Oakes S Luben R Khaw KT Bingham S Welch A Wareham N EPIC-Norfolk: study design and characteristics of the cohort Br J Cancer 1999 13 1 95 101 10466767

[B31] Stamp GH The appropriation of the parental role through communication during the transition to parenthood Comm Monogr 1994 13 2 89 112 10.1080/03637759409376327

[B32] Morris SM Joint and individual interviewing in the context of cancer Qual Health Res 2001 13 4 553 567 10.1177/104973201129119208 11521611

[B33] Brannen J Research note the study of sensitive subjects Sociol Rev 1988 13 3 552 563 10.1111/j.1467-954X.1988.tb02929.x

[B34] Valentine G Doing household research: interviewing couples together and apart Area 1999 13 1 67 74 10.1111/j.1475-4762.1999.tb00172.x

[B35] Edgell S Middle-Class Couples: a study of segregation, domination and inequality in marriage 1980 London: Allen and Unwin

[B36] Seymour J Dix G Eardley T Joint accounts Methodology and practice in research interviews with couples, Volume 5 1995 York: Social Policy Research Unit

[B37] Arksey H Collecting data through joint interviews Soc Res Update 2000 13 1 1 8

[B38] Milne J Oberle K Enhancing rigor in qualitative description J Wound Ostomy Continence Nurs 2005 13 6 413 420 10.1097/00152192-200511000-00014 16301909

[B39] Qualitative research review guidelines – RATShttp://www.biomedcentral.com/ifora/rats

[B40] Bryman A Hardy MA Handbook of data analysis 2009 London: Sage Publications Ltd

[B41] Morgan DL Qualitative content analysis: a guide to paths not taken Qual Health Res 1993 13 1 112 121 10.1177/104973239300300107 8457790

[B42] Morse JM Field PA Qualitative research methods for health professionals 1995 2 London: Sage Publications, Inc

[B43] Clark J F G, T J How to peer review a qualitative manuscript Peer Review in Health Sciences 2003 2 London: BMJ Books 219 235

[B44] Beck F Gillison F Standage M A theoretical investigation of the development of physical activity habits in retirement Br J Health Psychol 2010 13 3 663 679 10.1348/135910709X479096 19922724

[B45] Scanlon-Mogel JM Roberto KA Older adults’ beliefs about physical activity and exercise: life course influences and transitions Qual Ageing Older Adults 2004 13 3 33 44 10.1108/14717794200400017

[B46] Witcher CS Holt NL Spence JC Cousins SO A case study of physical activity among older adults in rural Newfoundland, Canada J Aging Phys Act 2007 13 2 166 183 17556783 10.1123/japa.15.2.166

[B47] Hirvensalo M Lintunen T Life-course perspective for physical activity and sports participation Eur Rev Aging Phys Act 2011 13 1 13 22 10.1007/s11556-010-0076-3

[B48] Strobl H Brehm W Tittlbach S Physical activity during the transition period between occupation and retirement Z Gerontol Geriatr 2010 13 5 297 301 10.1007/s00391-010-0103-z 20361195

[B49] Janzen W O’Brien Cousins S I do” or don’t: marriage, women, and physical activity throughout the lifespan J Women Aging 1995 13 1/2 55 70

[B50] Satariano WA Haight TJ Tager IB Living arrangements and participation in leisure-time physical activities in an older population J Aging Health 2002 13 4 427 451 10.1177/089826402237177 12391994

[B51] Snyder EE Spreitzer E Family influence and involvement in sports Res Q 1973 13 31 249 255

[B52] Falba TA Sindelar JL Spousal concordance in health behavior change Health Serv Res 2008 13 1 96 116 18211520 10.1111/j.1475-6773.2007.00754.x PMC2323137

[B53] Franks MM Shields CG Lim E Sands LP Mobley S Boushey CJ I will if you will similarity in married partners’ readiness to change health risk behaviors Health Educ Behav 2012 13 3 324 331 10.1177/1090198111402824 21498801

[B54] Padula C Older couples’ decision making on health issues West J Nurs Res 1996 13 6 675 687 10.1177/019394599601800605 9000874

[B55] Waite L Gallagher M The case for marriage: Why married people are healthier, happier, and better-off financially 2000 New York, NY: Doubleday

[B56] Ayotte BJ Margrett JA Hicks-Patrick J Physical activity in middle-aged and young-old adults J Health Psychol 2010 13 2 173 185 10.1177/1359105309342283 20207661

[B57] Resnick B A prediction model of aerobic exercise in older adults living in a continuing-care retirement community J Aging Health 2001 13 2 287 310 10.1177/089826430101300207 11787516

[B58] Williams GC Lynch MF McGregor HA Ryan RM Sharp D Deci EL Validation of the“ important other” climate questionnaire: assessing autonomy support for health-related change Fam Syst Health 2006 13 2 179 194

[B59] Hong TB Franks MM Gonzalez R Keteyian SJ Franklin BA Artinian NT A dyadic investigation of exercise support between cardiac patients and their spouses Health Psychol 2005 13 4 430 445 16045379 10.1037/0278-6133.24.4.430

[B60] Richards H Emslie C The ‘doctor’or the ‘girl from the University’? Considering the influence of professional roles on qualitative interviewing Fam Pract 2000 13 1 71 75 10.1093/fampra/17.1.71 10673494

