# Supplementary material for: How do couples influence each other’s physical activity behaviours in retirement? An exploratory qualitative study
Source: BMC Public Health. 2013 Dec 18;13:1197. doi: 10.1186/1471-2458-13-1197 (PMC3882289; doi:10.1186/1471-2458-13-1197)
Supplement: Additional file 1 — Topic guide for interview: physical activity of couples in retirement. [file 1471-2458-13-1197-S1.doc]

**Topic Guide for interview:**

**Physical activity of couples in retirement**

**1. Perceptions of physical activity across retirement**

- When you think of ‘physical activity’ what comes to mind?
- What does it mean for you to be ‘physically active’?
- What would you say ‘counts’ as physical activity?

Prompt, if necessary: Are ‘gardening’, walking, playing with grandchildren(?) and etc. physical activity?

- Do you do any physical activity or exercise nowadays?

Prompt if necessary: What activities? How often? How long? With

whom/alone? Is it part of a regular routine?

- Do you do any physical activity together as a couple? Can you describe?

Prompt if necessary: example going for walks, bike tours,

swimming. How regularly are you active together?

- Are you both retired nowadays? Please give details

Prompt if necessary: Since when? Fully retired or some work?

- What role does physical activity play in your life after retirement?

Probe after some time if necessary: How often are you active? How important is it for you?

- Would you say your physical activity behaviour has changed since you/your partner retired? If yes, how and why? Can you describe?

**2. Perceptions of physical activity across married life**

- Tell me about your physical activity patterns across your married life together? Were there any changes? Why?

Prompt, if necessary: Where there periods when one or both

partners’ physical activity level/type/intensity/duration increase

or decrease (for example, parenthood, job change, house

move)? Why?

- What role would you say has physical activity played across your married life?

Prompt if necessary: have you done physical activity together

(for example, going for walks, bike tours)

**3. Spousal interactions and influences on physical activity**

**Questions for couple:**

- Who would you say is more physical active in your partnership? Why?
- Do you feel you influence each others physical activity behaviour? How? Did it change after one/both of you retired?

Prompt if necessary: do you encourage each other to be

physically active? Did you introduce each other to certain activities?

**Questions for each spouse:**

- Do you think your partners’ attitude towards physical activity influence your own behaviour? Can you describe?
- Does your partner sometimes comment on your physical activity behaviours? How?

Prompt if necessary: Do you feel encourage by your partner be physical active? How? Or, why not?

- In what way, do your partners’ comments influence your own physical activity behaviour? Can you give details?
- Do you comment on your partners’ physical activity behaviour? How?
- Do you think your comments influence your partner’s physical activity behaviour?
- Do you think your partner’s activities influence your own physical activity behaviour?
- Do you think your activities influence your partner’s physical activity behaviour?
